# Eat Well, Sleep Well: Exploring the Association Between Eating Behavior and Sleep Quality

**DOI:** 10.3390/nu17172908

**Published:** 2025-09-08

**Authors:** Andrea Bazzani, Ugo Faraguna

**Affiliations:** 1 Institute of Management, Sant’Anna School of Advanced Studies, 56127 Pisa, Italy; 2 Department of Translational Research and of New Surgical and Medical Technologies, University of Pisa, 56126 Pisa, Italy; ugo.faraguna@unipi.it; 3 Department of Developmental Neuroscience, Istituto di Ricovero e Cura a Carattere Scientifico (IRCCS) Foundation Stella Maris, Calambrone, 56128 Pisa, Italy

## 1. Introduction

Eating and sleeping are two vital physiological functions occupying a significant portion of our daily routines and influencing numerous preparatory behaviors. Although mutually exclusive in time—we cannot eat while sleeping nor sleep while we eat under normal conditions [1]—they are deeply intertwined.

Insufficient or poor-quality sleep can directly affect food preferences, increasing the craving for calorie-rich or highly processed foods (e.g., [2]). For instance, two nights of sleep restriction reduce leptin levels and increase ghrelin levels, as well as hunger [3]. Moreover, sleep deprivation is associated with an increased appetite for sweets and high-calorie foods [3,4], likely reflecting a combination of reduced frontal cortex activity—highly sensitive to sleep loss—and increased amygdala activation [4].

Conversely, both the type and timing of food intake can impact sleep duration and quality [5]. Regular consumption of high-carbohydrate foods has been characterized by a shorter time to fall asleep, less deep sleep, and a higher proportion of REM sleep, whereas diets rich in fat have been linked to reduced efficiency of sleep but increased slow-wave sleep. Evidence also suggests that specific foods, including milk, fish, fruit, and vegetables, might promote sleep [6].

In general, healthy food intake is associated with better sleep quality, while processed and free-sugar-rich foods impair it [7]. In particular, dietary sources of tryptophan and melatonin showed sleep-promoting effects [8]. Eating close to bedtime also negatively impacts sleep quality [9], as stimulant-containing beverages and foods do [10]. Regular consumption of caffeine (and caffeine-containing drinks and foods), commonly used to counteract daytime sleepiness, has been linked to longer time to fall asleep, shorter total sleep duration, and reduced efficiency of sleep, and it can degrade sleep quality, particularly by diminishing slow-wave (deep) sleep. These effects are well documented across age groups and may be especially pronounced in older adults [11,12]. In adolescents, shorter sleep duration has been consistently linked to greater consumption of sugar-sweetened beverages and energy drinks, a pattern observed in multiple populations [13,14,15,16]. A recent study found that the effects of caffeine on sleep depend on both the dose and the timing of administration, with a 100 mg dose having no significant impact on sleep up to 4 h before bedtime, while a 400 mg dose negatively affects sleep when consumed anytime within 12 h of bedtime [17].

Alcohol, another widely used psychoactive substance, produces a paradoxical sleep profile: it may facilitate sleep initiation immediately after ingestion but tends to fragment sleep later in the night, alter normal architecture (particularly decreasing REM sleep during the latter part of the night), and increase awakenings and non-restorative sleep [18].

Although sleep and nutrition do not occur simultaneously, both follow cyclical patterns over a 24 h period. Their rhythm is circadian [19,20], placing them squarely within the field of chronobiology [21]. For instance, “time-restricted eating” emphasizes attention to limited time windows of food consumption to optimize fat loss [22]. This has given rise to the emerging field of chrononutrition, which links meal timing to circadian biology [23] and offers a fresh perspective on the potential synergy between sleep and eating in maintaining the body’s energy balance.

Sleep might be viewed as a downregulation of internal energy expenditure, while nutrition provides the external energy supply. During wakefulness, hypothalamic circuits coordinate somatic, autonomic, and endocrine functions to meet behavioral demands [24,25]. Conversely, the transition to sleep is marked by a reduction in metabolic rate [25]. This is reflected by whole-body indirect calorimetry, which shows a significant drop in human metabolism during sleep [26,27]. This reduction in energy use is partly mediated by central thermoregulatory downshifts, which conserve energy and mitigate environmental stress by lowering the body’s core temperature set point [25,26,28].

Since both eating and sleeping are governed by circadian rhythms [19,20], the degree of alignment between an individual’s sleep–wake cycle and food intake may underlie many of the observed health and behavioral outcomes. The relationship between the timing of food intake and chronotype, i.e., the preference for the timing of daily activities [29,30], provides a useful framework for understanding such an alignment. In a study conducted during the first COVID-19 lockdown [31], evening-type participants delayed both their main meals and bedtimes compared to the period before the lockdown. Freed from rigid social schedules, those with later chronotypes and those reporting poor sleep quality were more likely to shift their eating habits toward their intrinsic preferences, expressing a desire for a wider daytime eating window and more sleep opportunity overall.

Traditional nutritional guidance emphasizes a substantial early-day caloric intake [32], regular breakfast consumption [33], and an early dinner [34], often coupled with time-restricted eating that narrows the daily eating window [35]. Although these recommendations generally reduce cardiometabolic risk and improve sleep on average [36], they seldom account for individual chronotype differences. Evening chronotypes, for instance, are more likely to skip breakfast [37], postpone meals [38], and engage in nocturnal eating [39]—behaviors linked to poor sleep quality as well [9,40]. Lockdown data suggest that such behaviors become especially problematic when they exacerbate a mismatch between an individual’s preferred and actual meal-sleep timing [31].

## 2. Highlights from the Contributions of the Special Issue Studies

In this Special Issue, we welcomed scientific contributions adopting various methodological approaches to explore the interplay between sleep parameters and eating behavior. The eight published articles primarily focused on human models, evidencing a wide range of research and policy implications.

Al-Hinai and colleagues [41] used phone-survey data from 379 midlife Mexican women exploring meal timing and sleep (duration, latency, and quality), finding that later mealtimes and having less time between the last meal and bedtime were both independently associated with taking longer to fall asleep and reporting lower sleep quality, whereas findings regarding sleep duration were inconsistent.

In a large U.S. sample (N = 5228), assessing chrononutrition data with two 24 h recalls, Kim et al. [42] report that each additional hour between wake time and the first eating occasion, as well as between the last eating episode and bedtime, increased the likelihood of suboptimal sleep timing and duration, with the “Later Heavy Eating” and “Restricted Window Eating” chrononutrition phenotypes carrying the highest risk. These findings align with earlier work linking chronotype and sleep quality to broader nutritional health behaviors [43,44].

Vézina-Im et al. [45] extend this picture to adolescents (218 French-speaking adolescents (14–17 y) in Québec), documenting gender-specific associations between beverage consumption and sleep quality: energy drink intake was most detrimental for boys, whereas sugar-sweetened coffee had a stronger negative impact on girls’ sleep.

A specific target for intervention was proposed by Kosendiak et al. [46]. They followed first-year medical students, 570 students in the first months and 705 in the last part of the first academic year, and observed that: (i) two-thirds of students reported poor sleep at both timepoints; (ii) those with greater nutritional knowledge experienced less deterioration in sleep quality and used sleep medication less frequently over the academic year, suggesting a positive behavioral spillover between healthy eating literacy and sleep hygiene.

The positive effects of sleep hygiene and nutritional knowledge on sleep outcomes are confirmed in a sample of 402 young adults by Pokarowski and colleagues [47]. They also note that supplement and medication use are associated with worse sleep.

The two reviews in the Special Issue add mechanistic and life-course perspective.

A meta-analysis including 28 longitudinal studies conducted by Grimaldi and colleagues [48] further clarifies the overlooked relationship between sleep and eating behaviors in adolescents. According to their findings, longer, higher-quality sleep and fewer insomnia symptoms are related to lower BMI and fat percentage, while sleeping less than 7 h or experiencing poor sleep quality is tied to a higher risk of obesity. Yet they stress the shortage of longitudinal, bidirectional studies linking sleep timing and eating behaviors in adolescents.

From the perspective of sleep–wake pattern interaction with metabolic control, the work of Mogavero and colleagues [49] has thoroughly re-examined orexinergic signaling as a shared regulator of REM sleep and appetite in physiological states as well as in sleep-related disorders. The paper integrates clinical phenotypes (narcolepsy, sleep-related eating disorders) and suggests orexin pathways as promising therapeutic targets for sleep-metabolism comorbidity.

Finally, a promising randomized crossover protocol (planned n = 24) outlined in this Special Issue by Saidi et al. [50] will aim to compare diet-, exercise-, and mixed-induced acute energy deficits on sleep architecture (ambulatory polysomnography) and next-morning appetitive and reward responses.

Taken together, these five empirical studies, two reviews, and one protocol indicate that meal timing, specific dietary choices (including beverages), and nutritional/sleep knowledge all contribute to multidimensional sleep health; that these relationships are moderated by individual factors; and that advancing the field will require integrated approaches testing personalized interventions alongside population-level policies in longitudinal and mechanistic study designs.

## 3. Implications for Health Promotion

At the individual level, interventions that enhance healthy sleep and eating routines may work synergistically (e.g., [51]). Promoting regular sleep patterns—now shown to mitigate metabolic risk [52]—and reducing late-night behaviors such as bedtime procrastination [53] should be complemented by dietary guidance tailored to individual differences in sleep–wake patterns.

Where behavioral modification alone falls short, melatonin supplementation may help stabilize circadian rhythms [54,55].

Wearable devices, like actigraphy [56], may offer a powerful, non-invasive means of continuously monitoring both sleep regularity and related lifestyle behaviors, and their integration with diet logs can enhance our ability to track intervention efficacy in real time [57,58].

At the population level, educational campaigns should target specific at-risk groups, including evening chronotypes and adolescents, whose temporal preferences shape how they eat. Evening chronotypes show lower food-literacy skills, such as meal planning, budget-conscious shopping, and label interpretation, relying more on food availability cues and resulting in a higher body mass index compared to other chronotypes [59].

Drawing on insights from neuroeconomic models of food choice [60], public health programs can be designed to build label-reading and meal-planning competence while advocating for chronotype-aligned work and school schedules. Such an integrated approach promises to maximize benefits for sleep, metabolic health, and overall well-being.

## 4. A Key to Reading

Recent work in Nature has proposed a cellular mechanism defining the link between mitochondrial electron surplus and sleep needs [61]. In the fruit fly, the need for sleep appears to be a consequence of aerobic metabolism, as sleep deprivation in dorsal fan-shaped body neurons (dFBNs) leads to a mismatch between electron supply and ATP demand, causing mitochondrial morphological changes and heightened neuronal excitability that are reversed by sleep.

These emerging insights, from cell-level understanding to population-level implications, prompt us to consider the societal stakes of physiological interactions. Misaligned daily schedules, workplace demands, and recreational environments that promote unhealthy dietary habits and chronic sleep restriction can trigger a harmful vicious cycle. The public health burden of these dynamics is considerable, reflected by the growing rates of sleep disorders and unhealthy dietary patterns characterizing modern societies. Addressing this challenge requires combining individual behavior-change strategies with systemic interventions through a multidisciplinary approach that bridges physiology and clinical science with the social sciences.

## References

[1] Northeast R.C., Vyazovskiy V.V., Bechtold D.A. (2020). Eat, sleep, repeat: The role of the circadian system in balancing sleep–wake control with metabolic need. Curr. Opin. Physiol..

[2] Cappuccio F.P., D’Elia L., Strazzullo P., Miller M.A. (2010). Sleep duration and all-cause mortality: A systematic review and meta-analysis of prospective studies. Sleep.

[3] Spiegel K., Tasali E., Penev P., Van Cauter E.V. (2004). Brief communication: Sleep curtailment in healthy young men is associated with decreased leptin levels, elevated ghrelin levels, and increased hunger and appetite. Ann. Intern. Med..

[4] Greer S.M., Goldstein A.N., Walker M.P. (2013). The impact of sleep deprivation on food desire in the human brain. Nat. Commun..

[5] Andreeva V.A., Perez-Jimenez J., St-Onge M.P. (2023). A systematic review of the bidirectional association between consumption of ultra-processed food and sleep parameters among adults. Curr. Obes. Rep..

[6] St-Onge M.P., Mikic A., Pietrolungo C.E. (2016). Effects of diet on sleep quality. Adv. Nutr..

[7] Godos J., Grosso G., Castellano S., Galvano F., Caraci F., Ferri R. (2021). Association between diet and sleep quality: A systematic review. Sleep Med. Rev..

[8] Zuraikat F.M., Wood R.A., Barragán R., St-Onge M.P. (2021). Sleep and diet: Mounting evidence of a cyclical relationship. Annu. Rev. Nutr..

[9] Crispim C.A., Zimberg I.Z., dos Reis B.G., Diniz R.M., Tufik S., de Mello M.T. (2011). Relationship between food intake and sleep pattern in healthy individuals. J. Clin. Sleep Med..

[10] Sejbuk M., Mirończuk-Chodakowska I., Witkowska A.M. (2022). Sleep quality: A narrative review on nutrition, stimulants, and physical activity as important factors. Nutrients.

[11] Clark I., Landolt H.P. (2017). Coffee, caffeine, and sleep: A systematic review of epidemiological studies and randomized controlled trials. Sleep Med. Rev..

[12] Robillard R., Bouchard M., Cartier A., Nicolau L., Carrier J. (2015). Sleep is more sensitive to high doses of caffeine in the middle years of life. J. Psychopharmacol..

[13] Sampasa-Kanyinga H., Hamilton H.A., Chaput J.P. (2018). Sleep duration and consumption of sugar-sweetened beverages and energy drinks among adolescents. Nutrition.

[14] Lohsoonthorn V., Khidir H., Casillas G., Lertmaharit S., Tadesse M.G., Pensuksan W.C., Rattananupong T., Gelaye B., Williams M.A. (2013). Sleep quality and sleep patterns in relation to consumption of energy drinks, caffeinated beverages, and other stimulants among Thai college students. Sleep Breath..

[15] Prather A.A., Leung C.W., Adler N.E., Ritchie L., Laraia B., Epel E.S. (2016). Short and sweet: Associations between self-reported sleep duration and sugar-sweetened beverage consumption among adults in the United States. Sleep Health.

[16] Sanchez S.E., Martinez C., Oriol R.A., Yanez D., Castañeda B., Sanchez E., Gelaye B., Williams M.A. (2013). Sleep quality, sleep patterns and consumption of energy drinks and other caffeinated beverages among Peruvian college students. Health.

[17] Gardiner C.L., Weakley J., Burke L.M., Fernandez F., Johnston R.D., Leota J., Russell S., Munteanu G., Townshend A., Halson S.L. (2025). Dose and timing effects of caffeine on subsequent sleep: A randomized clinical crossover trial. Sleep.

[18] Ebrahim I.O., Shapiro C.M., Williams A.J., Fenwick P.B. (2013). Alcohol and sleep I: Effects on normal sleep. Alcohol. Clin. Exp. Res..

[19] Borbély A.A. (1982). A two process model of sleep regulation. Hum. Neurobiol..

[20] Challet E. (2019). The circadian regulation of food intake. Nat. Rev. Endocrinol..

[21] Kuhlman S.J., Craig L.M., Duffy J.F. (2018). Introduction to chronobiology. Cold Spring Harb. Perspect. Biol..

[22] Xie Y., Zhou K., Shang Z., Bao D., Zhou J. (2024). The effects of time-restricted eating on fat loss in adults with overweight and obese depend upon the eating window and intervention strategies: A systematic review and meta-analysis. Nutrients.

[23] Pot G.K. (2018). Sleep and dietary habits in the urban environment: The role of chrono-nutrition. Proc. Nutr. Soc..

[24] Thompson R.H., Swanson L.W. (2003). Structural characterization of a hypothalamic visceromotor pattern generator network. Brain Res. Rev..

[25] Parmeggiani P.L. (2003). Thermoregulation and sleep. Front. Biosci..

[26] Kräuchi K., de Boer T., Kryger M.H., Roth T., Dement W.C. (2011). Body Temperatures, Sleep, and Hibernation. Principles and Practice of Sleep Medicine.

[27] Katayose Y., Tasaki M., Ogata H., Nakata Y., Tokuyama K., Satoh M. (2009). Metabolic rate and fuel utilization during sleep assessed by whole-body indirect calorimetry. Metabolism.

[28] Harding E.C., Franks N.P., Wisden W. (2019). The temperature dependence of sleep. Front. Neurosci..

[29] Horne J.A., Östberg O. (1977). Individual differences in human circadian rhythms. Biol. Psychol..

[30] Adan A., Natale V. (2002). Gender differences in morningness–eveningness preference. Chronobiol. Int..

[31] Bazzani A., Marantonio S., Andreozzi G., Lorenzoni V., Bruno S., Cruz-Sanabria F., d’Ascanio P., Turchetti G., Faraguna U. (2022). Late chronotypes, late mealtimes. Chrononutrition and sleep habits during the COVID-19 lockdown in Italy. Appetite.

[32] Jakubowicz D., Barnea M., Wainstein J., Froy O. (2013). High caloric intake at breakfast vs. dinner differentially influences weight loss of overweight and obese women. Obesity.

[33] Uzhova I., Mullally D., Peñalvo J.L., Gibney E.R. (2018). Regularity of breakfast consumption and diet: Insights from national adult nutrition survey. Nutrients.

[34] Gu C., Brereton N., Schweitzer A., Cotter M., Duan D., Børsheim E., Wolfe R.R., Pham L.V., Polotsky V.Y., Jun J.C. (2020). Metabolic effects of late dinner in healthy volunteers—A randomized crossover clinical trial. J. Clin. Endocrinol. Metab..

[35] Chaix A., Manoogian E.N., Melkani G.C., Panda S. (2019). Time-restricted eating to prevent and manage chronic metabolic diseases. Annu. Rev. Nutr..

[36] Almoosawi S., Vingeliene S., Gachon F., Voortman T., Palla L., Johnston J.D., Van Dam R.M., Darimont C., Karagounis L.G. (2019). Chronotype: Implications for epidemiologic studies on chrono-nutrition and cardiometabolic health. Adv. Nutr..

[37] Meule A., Roeser K., Randler C., Kübler A. (2012). Skipping breakfast: Morningness–eveningness preference is differentially related to state and trait food cravings. Eat. Weight Disord..

[38] Reutrakul S., Hood M.M., Crowley S.J., Morgan M.K., Teodori M., Knutson K.L., Van Cauter E.V.E. (2013). Chronotype is independently associated with glycemic control in type 2 diabetes. Diabetes Care.

[39] Kandeger A., Egilmez U., Sayin A.A., Selvi Y. (2018). The relationship between night eating symptoms and disordered eating attitudes via insomnia and chronotype differences. Psychiatry Res..

[40] Cheng S.H., Shih C.C., Lee I.H., Hou Y.W., Chen K.C., Chen K.T., Yang Y.K., Yang Y.C. (2012). A study on the sleep quality of incoming university students. Psychiatry Res..

[41] Al-Hinai M., Mohy A., Téllez-Rojo M.M., Torres-Olascoaga L.A., Bautista-Arredondo L.F., Cantoral A., Peterson K.E., Jansen E.C. (2024). Meal Timing and Sleep Health Among Midlife Mexican Women During the Early Stages of the COVID-19 Pandemic. Nutrients.

[42] Kim N., Conlon R.K., Farsijani S., Hawkins M.S. (2024). Association between chrononutrition patterns and multidimensional sleep health. Nutrients.

[43] Muscogiuri G., Barrea L., Aprano S., Framondi L., Di Matteo R., Laudisio D., Pugliese G., Savastano S., Colao A., Opera Prevention Project (2020). Chronotype and adherence to the Mediterranean diet in obesity: Results from the Opera Prevention Project. Nutrients.

[44] Bruno S., Bazzani A., Marantonio S., Cruz-Sanabria F., Benedetti D., Frumento P., Turchetti G., Faraguna U. (2022). Poor sleep quality and unhealthy lifestyle during the lockdown: An Italian study. Sleep Med..

[45] Vézina-Im L.-A., Beaulieu D., Turcotte S., Turcotte A.-F., Delisle-Martel J., Labbé V., Lessard L., Gingras M. (2024). Association between beverage consumption and sleep quality in adolescents. Nutrients.

[46] Kosendiak A.A., Adamczak B.B., Kuźnik Z., Makles S. (2024). Impact of medical school on the relationship between nutritional knowledge and sleep quality—A longitudinal study of students at Wroclaw Medical University in Poland. Nutrients.

[47] Pokarowski M., Kedra M., Piwinska J., Kurek K., Szczygiel K., Denysiuk P., Popiolek-Kalisz J. (2023). The relationship between knowledge, dietary supplementation, and sleep quality in young adults after the COVID-19 pandemic. Nutrients.

[48] Grimaldi M., Bacaro V., Natale V., Tonetti L., Crocetti E. (2023). The longitudinal interplay between sleep, anthropometric indices, eating behaviors, and nutritional aspects: A systematic review and meta-analysis. Nutrients.

[49] Mogavero M.P., Godos J., Grosso G., Caraci F., Ferri R. (2023). Rethinking the Role of Orexin in the Regulation of REM Sleep and Appetite. Nutrients.

[50] Saidi O., Chatain C., Del Sordo G.C., Demaria R., Lequin L., Rochette E., Larribaut J., Gruet M., Duché P. (2023). The Effects of Different Modalities of an Acute Energy Deficit on Sleep and Next Morning Appetitive and Compensatory Behavior in Healthy Young Adults: The EDIES Protocol. Nutrients.

[51] Vera B., Dashti H.S., Gómez-Abellán P., Hernández-Martínez A.M., Esteban A., Scheer F.A.J.L., Saxena R., Garaulet M. (2018). Modifiable lifestyle behaviors, but not a genetic risk score, associate with metabolic syndrome in evening chronotypes. Sci. Rep..

[52] Huang T., Redline S. (2019). Cross-sectional and prospective associations of actigraphy-assessed sleep regularity with metabolic abnormalities: The Multi-Ethnic Study of Atherosclerosis. Diabetes Care.

[53] Kroese F.M., Evers C., Adriaanse M.A., de Ridder D.T. (2016). Bedtime procrastination: A self-regulation perspective on sleep insufficiency in the general population. J. Health Psychol..

[54] Cruz-Sanabria F., Carmassi C., Bruno S., Bazzani A., Carli M., Scarselli M., Faraguna U. (2023). Melatonin as a chronobiotic with sleep-promoting properties. Curr. Neuropharmacol..

[55] Cruz-Sanabria F., Bruno S., Crippa A., Frumento P., Scarselli M., Skene D.J., Faraguna U. (2024). Optimizing the time and dose of melatonin as a sleep-promoting drug: A systematic review of randomized controlled trials and dose–response meta-analysis. J. Pineal Res..

[56] Ancoli-Israel S., Cole R., Alessi C., Chambers M., Moorcroft W., Pollak C.P. (2003). The role of actigraphy in the study of sleep and circadian rhythms. Sleep.

[57] Barragán R., Zuraikat F.M., Tam V., Scaccia S., Cochran J., Li S., Cheng B., St-Onge M.-P. (2021). Actigraphy-derived sleep is associated with eating behavior characteristics. Nutrients.

[58] Rusu A., Ciobanu D.M., Inceu G., Craciun A.E., Fodor A., Roman G., Bala C.G. (2022). Variability in sleep timing and dietary intake: A scoping review of the literature. Nutrients.

[59] Trieste L., Bazzani A., Amato A., Faraguna U., Turchetti G. (2021). Food literacy and food choice–a survey-based psychometric profiling of consumer behaviour. Br. Food J..

[60] Plassmann H., Schelski D.S., Simon M.C., Koban L. (2022). How we decide what to eat: Toward an interdisciplinary model of gut–brain interactions. Wiley Interdiscip. Rev. Cogn. Sci..

[61] Sarnataro R., Velasco C.D., Monaco N., Kempf A., Miesenböck G. (2025). Mitochondrial origins of the pressure to sleep. Nature.

